# Supplementary material for: Bulk-local-density-of-state correspondence in topological insulators
Source: Nat Commun. 2023 Nov 14;14:7347. doi: 10.1038/s41467-023-42449-2 (PMC10646048; doi:10.1038/s41467-023-42449-2)
Supplement: Supplementary file 1 — Supplementary Information [file 41467_2023_42449_MOESM1_ESM.pdf]

Supplementary Information for Bulk Local-Density-of-State Correspondence in Topological Insulators

Biye Xie[1,5†], Renwen Huang[2,3†], Shiyin Jia[2,3†], Zemeng Lin[1†], Junzheng Hu[2,3], Yao Jiang[2,3], Shaojie Ma[1], Peng Zhan[2,3*], Minghui Lu[2,4*], Zhenlin Wang[2,3], Yanfeng Chen[2,4] and Shuang Zhang[1*]

[1]New Cornerstone Science Laboratory, Department of Physics, The University of Hong Kong, Pokfulam Road, Hong Kong, China.
[2]National Laboratory of Solid State Microstructures, Collaborative Innovation Center of Advanced Microstructures, Nanjing University, Nanjing, 210093, China.
[3]School of Physics, Nanjing University, Nanjing, 210093, China.
[4]Department of Materials Science and Engineering, Nanjing University, Nanjing, 210093, China.
[5]School of Science and Engineering, The Chinese University of Hong Kong, Shenzhen, 518172, China.

*Corresponding author(s). E-mail(s): zhanpeng@nju.edu.cn; luminghui@nju.edu.cn; shuzhang@hku.hk;
†These authors contributed equally to this work.

The Supplementary Information file includes:
Supplementary Text
Supplementary Figs. S1 to S7
Supplementary References (1 to 2)

Supplementary Note 1: Bulk-LDOS correspondence in tight-binding models

The photonic band structure and the topological properties of the isotropic photonic crystal can be well approximated by the tight-binding model (see Fig. S1a), whose Hamiltonian is as follows

$$H(\mathbf{k}) = \begin{pmatrix} 0 & h_{12} & h_{13} & 0 \\ h_{21} & 0 & 0 & h_{24} \\ h_{31} & 0 & 0 & h_{34} \\ 0 & h_{42} & h_{43} & 0 \end{pmatrix} \tag{1}$$

Here $h_{12} = t_a + t_b\exp(\mathrm{i}k_x)$, $h_{13} = t_a + t_b\exp(-\mathrm{i}k_y)$, $h_{21} = t_a + t_b\exp(-\mathrm{i}k_x)$, $h_{24} = t_a + t_b\exp(-\mathrm{i}k_y)$, $h_{31} = t_a + t_b\exp(\mathrm{i}k_y)$, $h_{34} = t_a + t_b\exp(\mathrm{i}k_x)$, $h_{42} = t_a + t_b\exp(\mathrm{i}k_y)$, $h_{43} = t_a + t_b\exp(-\mathrm{i}k_x)$. $\mathbf{k} = (k_x, k_y)$ is the Bloch momentum. The tight-binding parameters t_a, t_b reflect the intra- and inter-cell couplings between the neighboring rods, respectively. Here, we neglect the higher-order couplings such as the next-nearest-neighbor coupling. This approximation is valid except for the lowest band which will not change the topology of the band structure as shown in Fig. S1b-c. If the intercell coupling is larger than the intracell coupling, the tight-binding model is in topologically nontrivial insulating phase and there will be topological edge states and corner states under the open boundary condition [1]. We can also add perturbations on the onsite energy of edge lattice sites to 'drag' the ingap topological edge states into the bulk spectrum (see Fig. S1c). Here we choose $t_a = 1.05$, $t_b = 1.6$. When onsite energy perturbation $E_{Edge} = 1$ is added for the edge lattice of the finite chain [2], the edge states are now pulled from the middle of the directional bandgap into the bulk bands. Nevertheless we can observe a multidimensional partition of LDOS for topologically nontrivial phase which clearly demonstrate the bulk-LDOS correspondence for the tight-binding model.

To calculate the LDOS, we first obtain the eigenfunctions of real space lattices and corresponding eigenfrequencies by diagonalizing the Hamiltonian of the finite-size array. We can add up those wavefunctions by their eigenfrequencies to obtain the real space distribution of LDOS. We define the local density of states of the bulk, edge and corner region as depicted in Fig. S1d-i. According to the Figs. S1d-f, at the lower range of the eigenfrequencies, the eigenstates are nearly uniformly distributed at the whole lattice sites for any frequencies for the trival case. However, for the nontrivial lattice as shown in Figs. S1g-i, the LDOS of eigenstates is firstly distributed mainly at the bulk lattice sites, avoiding the edge and corner lattice sites, when the energy of states is low. As we increase the energy, the LDOS of eigenstates is mainly distributed at both the bulk and edge sites, avoiding the corner sites, representing the coexistence of bulk and edge states at this energy. Furthermore, as we continuously increase the energy of states, we find that the LDOS is now mainly distributed at both bulk and corner sites, avoiding edge sites which indicating the coexistence of both bulk and corner states at this energy. The above character in tight-binding models clearly reveals the multidimensional partitions of LDOS

for topologically non-trivial insulating phases which consists with the case in photonic crystals.

Next, we add random onsite energy perturbations to the whole lattices. As shown in Fig. S2, Even the disorder breaks the C_4 symmetry of the lattice and the LDOS is not symmetrized, we can also observe a similar multidimensional partition of LDOS for topologically nontrivial phase and a single-dimensional partition of LDOS for topologically trivial phase. Therefore the bulk-LDOS correspondence can distinguishing different topological phases in topological insulators characterized by the displacement of Wannier centers.

Supplementary Note 2:Experimental setup

We fabricate our photonic crystals by using Z_rO_2 rods with a dielectric constant $\epsilon = 28$. The height of the cylinders is 9.5mm for all samples. The lattice constant of the unit cell is 20mm. Each sample is made of an aluminum plate with dielectric pillars inserted into shallow holes of 0.5mm depth for fixation. A thin film of air outside the PC with a thickness of $0.25 \times a$ is introduced between the PCs and the aluminum frames(serve as perfect electric conduction boundaries) on each side of samples, as depicted in Fig. s3. For the measurement of the data, we averagely divide each unit cell to 10×10 parts and detect the central region of each part in a range of $2 - 9$GHz with a resolution about 5MHz (see Fig. s4).

Supplementary Note 3: The difference between excited field distribution and local density of states

In traditional bulk-boundary correspondence, the topologically nontrivial phase is revealed by the measurement of the excitation field distribution at the frequency of in-gap edge/corner states. However, for general systems with directional bandgaps, this approach fails since there is no in-gap states or bound-state in the continuum(BIC) and the excitation field will significantly influenced by the overlap between the source profile and the eigenstates. For example, we here consider three cases in topologically nontrivial phase:(1) The LDOS is distributed mainly at the bulk sites(see Fig. S5a). (2) The LDOS is distributed mainly at the bulk and edge sites(see Fig. S5e). (3) The LDOS is distributed mainly at the corner sites(see Fig. S5i). We find that the excited field distributions with different positions of a point source are significantly varying and thus cannot rigorously reveal the bulk topology (see Fig. S5).

Supplementary Note 4: The difference between topological boundary states and defective boundary localized states

Besides topological boundary states, defective edge localized states may also emerge by adding a large onsite energy potential to the boundary lattice sites in the two-dimensional systems. However, these kind of boundary localized defective states originate from the detune of boundary sites to the bulk sites resulting in several fundamental differences from the topological boundary state which are also reflected in their LDOSs. First, topological boundary states stem from re-hybridizations of multiple bands while the defective edge states come from the single band. As a consequence, as shown in Figs. s6a and s6c, the number of topological edge states and corner states are 2 and 1 respectively at each boundary (the total number in a finite square structure is 8 and 4 due to the four-fold rotational symmetry). However, when adding a large onsite energy potential to the boundary sites of a trivial lattice, as shown in Figs. s7a and s7b, the number of emergent edge and corner localized states are 4 and 3 respectively at different frequencies in the s-band for a specific M and therefore can be distinguished by looking at the spectrum of the LDOS. Secondly, The LDOSs for topological boundary state and defective boundary localized states are also different in real space. As shown in Figs. s6c (Fig. s7c), the LDOS for topological edge (corner) states is strongly localized at the edge (corner) sites and the sites belong to the same sublattice in the next-nearest edge (corner) cells. However, for defective edge (corner) localized states, the LDOSs are extended to other sites in the edge (corner) cells and do not confined in one sublattice. These difference between topological boundary states and defective boundary localized states in the LDOS at both energy domain and real space enable us to identify distinct topological phase by looking at the LDOS characters which is just like the cases with complete bandgaps [2]. We also verify these differences in photonic crystals which is consist with the tight binding models.

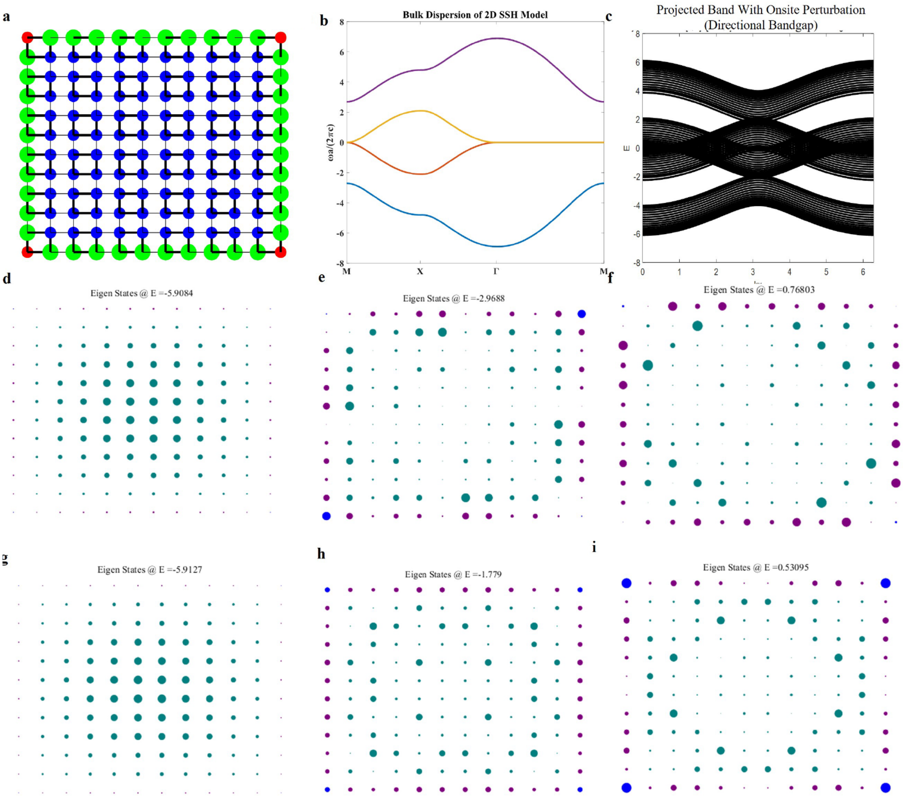

Fig. S1 Multidimensional partition of LDOS for topologically nontrivial tight-binding model. a The finite-size lattice with perturbed edge sites with directional bandgap and no in-gap states. **b** The bulk band structure with directional bandgap. **c** The projected band structure with no in-gap edge states.**d-f** The LDOS of topological lattice at energy $E = -5.9084$, $E = -2.9688$, and $E = 0.76803$ respectively. **g-i** The LDOS of trivial lattice at energy $E = -5.9127$, $E = -1.779$, and $E = 0.53095$ respectively.

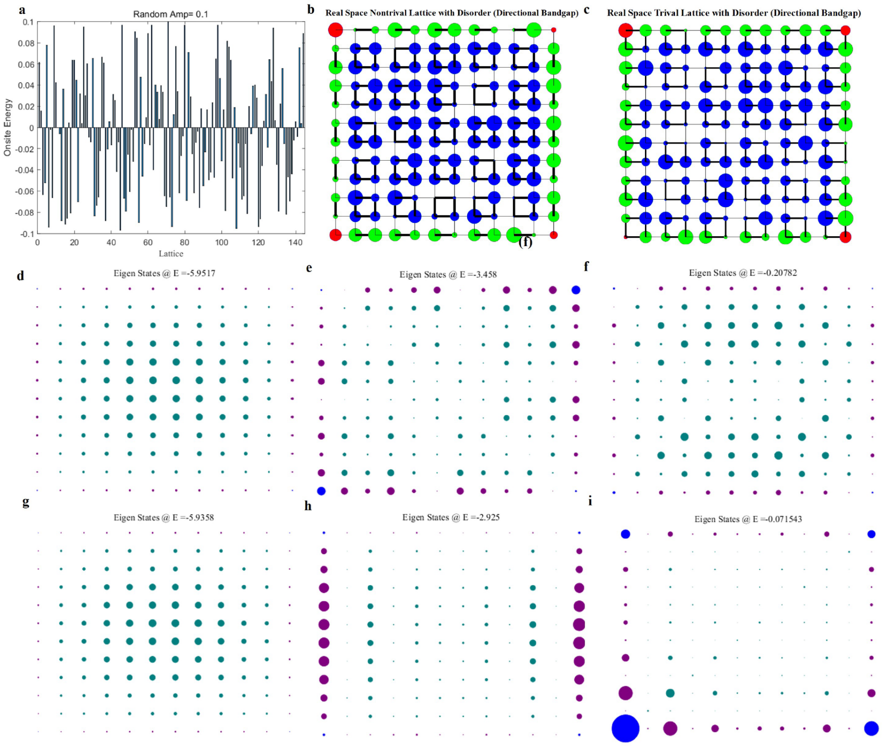

Fig. S2 Multidimensional partition of LDOS for topologically nontrivial tight-binding model with random disorders. a The added random onsite perturbation at each site. **b-c** The finite size lattice with random disorders for topologically trivial and nontrivial lattice. **d-f** The LDOS of topologically trivial lattice at energy $E = -5.9517$, $E = -3.458$, and $E = -0.20782$ respectively. **g-i** The LDOS of topologically non-trivial lattice at energy $E = -5.9358$, $E = -2.925$, and $E = -0.071543$ respectively.

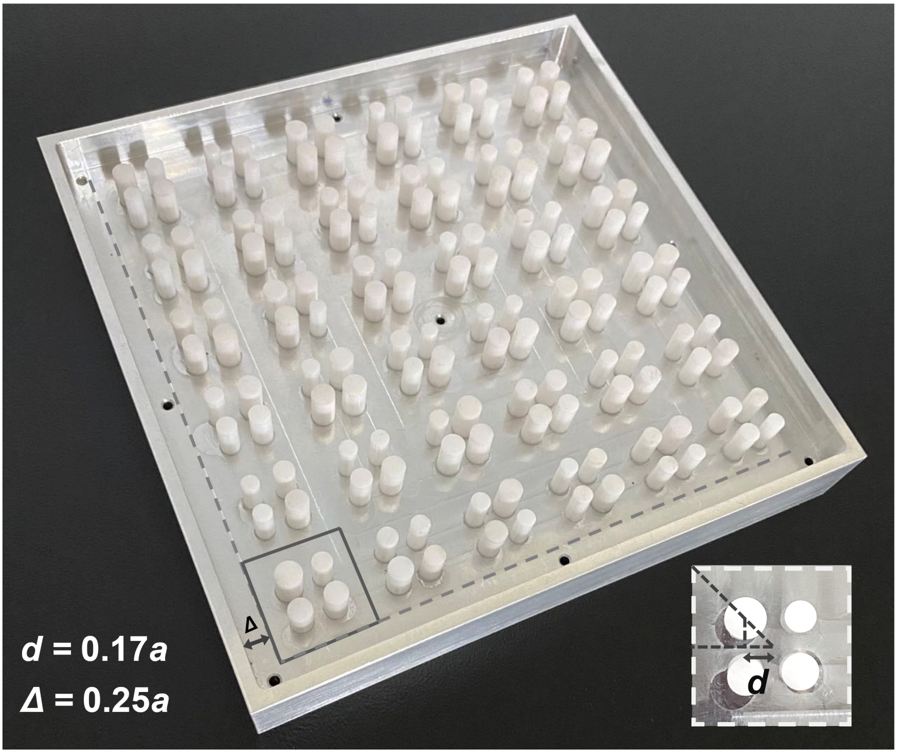

Fig. S3 Photograph of the sample. The boundary of PCs and the air gap is indicated as the dashed line, $\Delta = 0.25a$ is the thickness of the air gap. The lower right panel is a zoom-in picture of the unit cell where d denotes the distances of the dielectric cylinders from the center of the unit cell both horizontally and vertically. The picture shown here is the sample of $d = 0.17a$ with the onsite potential disorder.

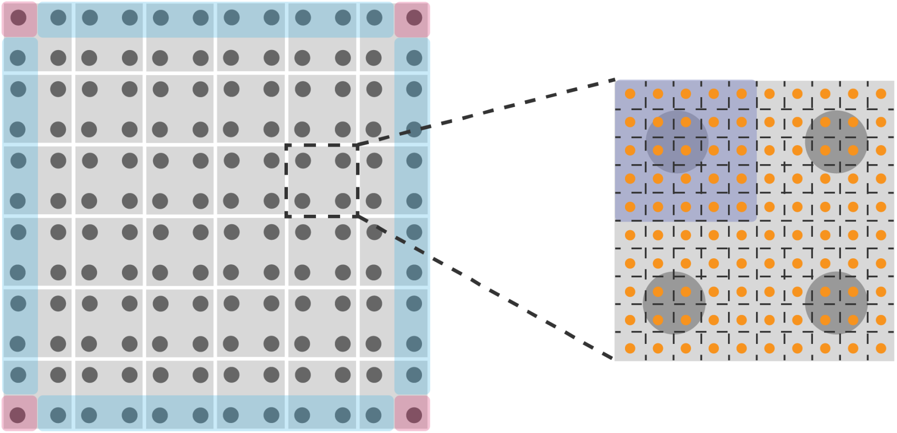

Fig. S4 Schematic of sampled points. Unit cells and the dielectric cylinders are represented as gray squares and darker circles. The blue and pink regions denote the edges and corners of the sample, respectively. Each unit cell is divided into 10×10 equivalent areas with orange dots locate at the center of each area which are the measured points.

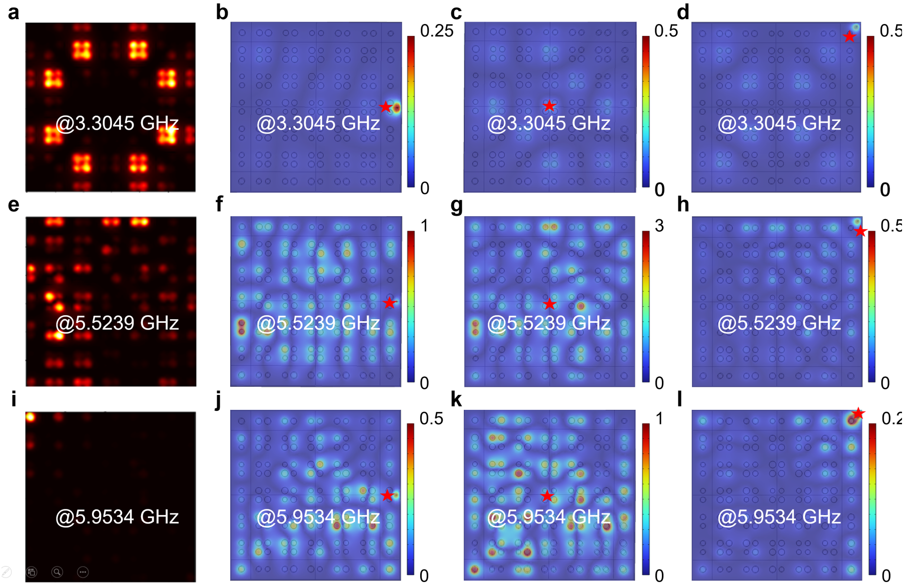

Fig. S5 The difference between the LDOSs and the excited field distributions. **a** The simulated LDOS of an eigenstate at $f = 3.3045$GHz. **b-d** The simulated excited field distributions at $f = 3.3045$GHz with a point source placed at the center of the side, the center of the bulk and the corner of the finite-size structure respectively. **e** The simulated LDOS of a eigenstate at $f = 5.5239$GHz. **f-h** The simulated excited field distributions at $f = 5.5239$GHz with a point source placed at the center of the side, the center of the bulk and the corner of the finite-size structure respectively. **i** The simulated LDOS of a eigenstate at $f = 5.9534$GHz. **j-l** The simulated excited field distributions at $f = 5.9534$GHz with a point source placed at the center of the side, the center of the bulk and the corner of the finite-size structure respectively.

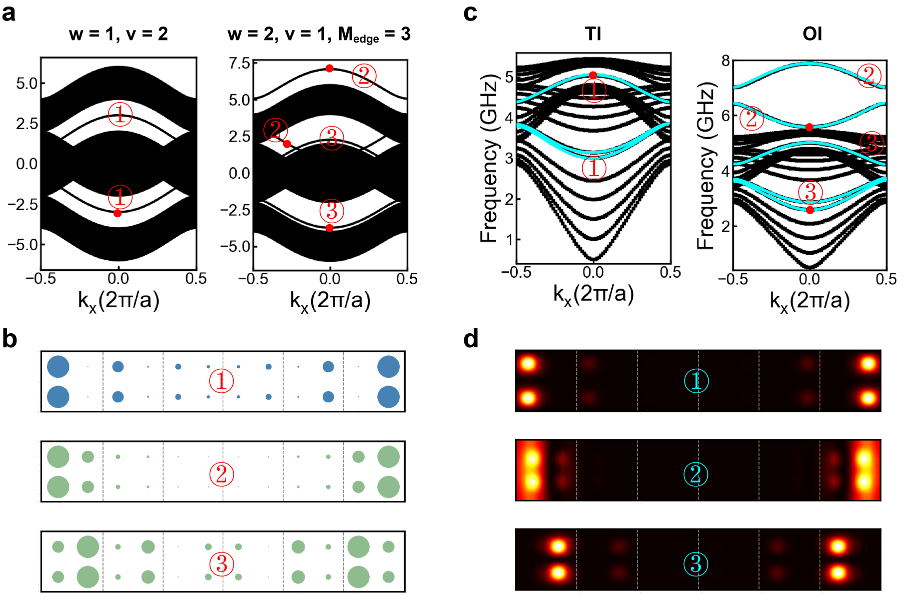

Fig. S6 The difference between the defective edge states and topological edge states. **a** The projective band structures of the tight-binding models for topological edge states (represented by number 1) with intracell coupling $\omega = 1$ and intercell coupling $v = 2$, and the defective edge states (represented by number 2 and 3) induced by adding large onsite potential ($M_{edge} = 1$) on the edge sites of trivial lattice ($\omega = 2$, and $v = 1$). **b** The local density of states (LDOS) for topological edge states (represented by number 1) and defective edge states (represented by number 2 and 3). **c** Numerical calculated projective band structures of the photonic crystals for the topological edge states (represented by number 1) and the defective edge states (represented by number 2 and 3) which are similar to the tight-binding models. **d** LDOS for photonic topological edge states (represented by number 1) and defective edge states (represented by number 2 and 3).

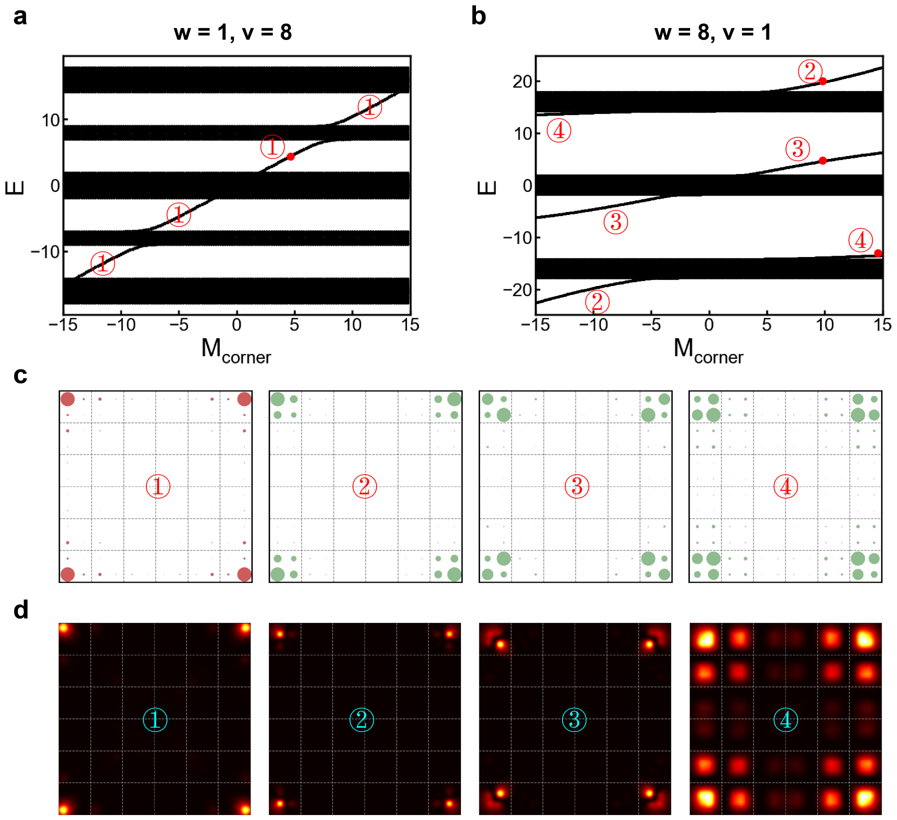

Fig. S7 The difference between the defective corner states and topological corner states a a The evolution of eigenmodes of the topological corner states (represented by number 1) and **b** the defective edge states (represented by number 2 and 3, 4) in a trivial lattice under the change of different onsite energy potential (M_{corner}) on the four corner sites. **c** LDOS for topological corner states is strongly localized at the corner sites (marked by 1); LDOSs for defective corner localized states are extended to other sites near the corner sites at the corner unit cells (marked by 2, 3, and 4). **d** Calculated LDOSs for similar case as those in (c) in photonic crystals.

References

[1] Xie, B. Y., Su, G. X., Wang, H. F., Su, H., Shen, X. P., Zhan, P., Lu, M. H., Wang, Z. L. & Chen, Y. F. *Phys. Rev. Lett.* **122**, 233903 (2019).

[2] Peterson, C. W., Li, T., Benalcazar, W. A., Hughes, T. L. & Bahl, G., *Science*, **368**, 1114-1118 (2020).
